# Temporal Dynamics of Visual Attention Measured with Event-Related Potentials

**DOI:** 10.1371/journal.pone.0070922

**Published:** 2013-08-19

**Authors:** Yoshiyuki Kashiwase, Kazumichi Matsumiya, Ichiro Kuriki, Satoshi Shioiri

**Affiliations:** 1 Graduate School of Information Sciences, Tohoku University, Sendai, Japan; 2 Research Institute of Electrical Communication, Tohoku University, Sendai, Japan; 3 Japan Society for the Promotion of Science, Tokyo, Japan; Nothwestern University, United States of America

## Abstract

How attentional modulation on brain activities determines behavioral performance has been one of the most important issues in cognitive neuroscience. This issue has been addressed by comparing the temporal relationship between attentional modulations on neural activities and behavior. Our previous study measured the time course of attention with amplitude and phase coherence of steady-state visual evoked potential (SSVEP) and found that the modulation latency of phase coherence rather than that of amplitude was consistent with the latency of behavioral performance. In this study, as a complementary report, we compared the time course of visual attention shift measured by event-related potentials (ERPs) with that by target detection task. We developed a novel technique to compare ERPs with behavioral results and analyzed the EEG data in our previous study. Two sets of flickering stimulus at different frequencies were presented in the left and right visual hemifields, and a target or distracter pattern was presented randomly at various moments after an attention-cue presentation. The observers were asked to detect targets on the attended stimulus after the cue. We found that two ERP components, P300 and N2pc, were elicited by the target presented at the attended location. Time-course analyses revealed that attentional modulation of the P300 and N2pc amplitudes increased gradually until reaching a maximum and lasted at least 1.5 s after the cue onset, which is similar to the temporal dynamics of behavioral performance. However, attentional modulation of these ERP components started later than that of behavioral performance. Rather, the time course of attentional modulation of behavioral performance was more closely associated with that of the concurrently recorded SSVEPs analyzed. These results suggest that neural activities reflected not by either the P300 or N2pc, but by the SSVEPs, are the source of attentional modulation of behavioral performance.

## Introduction

Visual attention is a brain function that selects potentially important information from a vast amount of incoming sensory information. Optimal scene perception often requires the shifting of visual attention to various locations in a scene, and the time course of these shifts is one of the most important factors for the function. Temporal dynamics have been reported to differ between different types of attention (e.g., exogenous and endogenous attention), and the temporal properties of the shifts in visual attention could therefore help us to understand the mechanisms underlying the function.

The time course of attention shifts has been investigated psychophysically and physiologically in a variety of conditions. A typical measurement method is a pre-cue paradigm [1], [2]. In this paradigm, a pre-cue informs the participants about the location of an upcoming target some time before the target is presented. Behavioral performance improves with time after cueing within a period of several tens or hundreds milliseconds (see reviews by [1], [3]). A similar time course of attention shift has been seen for physiological measurements of steady-state visual evoked potentials (SSVEPs). SSVEPs are oscillatory electroencephalogram (EEG) potentials that occur synchronously to flickering visual stimuli. The magnitude of a visual response to a flickering stimulus can be tracked by the flicker frequency in the EEG signal [4], [5]. Müller et al. [6] measured the time course of attentional modulation by tracking SSVEP amplitude in time and estimated that it takes 600–800 ms for cortical facilitation by visual attention (see also [7], [8], [9]).

An important question has been raised regarding how the results measured psychophysically are related to those measured physiologically [6]. One way to investigate the relationship between psychophysical and physiological data is to compare their temporal characteristics of attentional modulation. The time-course comparison could help to understand whether the measures reflect same or different mechanisms (e.g., [10]) or which measure causally determine the other measure (e.g., [11]). Psychophysical measurements of attention shifts are expressed with the time of stimulus presentation relative to the cue presentation as an independent variable, whereas physiological measurements are expressed with the time of brain activity relative to the cue presentation. Since there is a delay between stimulus presentation and the brain activities evoked by the stimulus [12], psychophysical measurements corresponding to the stimulus should not be related to brain activity at the time of retinal stimulation [6]. Instead, the brain activity corresponding to a stimulus should be obtained after some delay. Therefore, to compare the time course of the physiological data with that of the behavioral data, it is necessary to assume a time lag between the onsets for neural activities and behavioral performance. Previous studies assumed that the delay was 100–150 ms and evaluated the relations between behavioral and physiological data by subtracting the delay from the latency of attentional modulation of physiological measures [6], [8]. In this study, we developed a novel method, which allows direct comparison of temporal properties between psychophysical and physiological results without making any assumption on the time lag between these measures. To compare the behavioral and physiological data, we used some landmarks in the event-related potentials (ERPs), which are known to be sensitive measures of visual attention, such as P300 and N2pc components. By evaluating the temporal modulation of those characteristic peak components as a function of stimulus onset latency, it became possible to compare the attentional time courses directly between both measures, without assuming the lags between the measures.

Generally, amplitudes of ERPs to the attended visual stimulus are larger than those to the ignored stimulus. To our knowledge, no study has compared the time course of attention shift between behavioral and ERP data directly. Most of the previous studies of the time course of attention have investigated the changes in each ERP component to a single target (e.g., [13], [14]), not the changes in ERP amplitude after cueing to shift attention. When we measure ERPs to targets presented after cueing, the ERP for each target can be expressed as a function of target-presentation latency after the cue presentation, as well as the behavioral results. This enables us to compare the behavioral data directly with the physiological data. If we can compare ERP measurements with behavioral performance, we can investigate which component of the evoked potential determines subjective perception. Several ERP components have been suggested to be related to attention. For example, Luck et al. found that the temporal dynamics of behavioral performance during attentional blink are correlated to those of P3 amplitude but not of P1, N1, and N400 amplitudes [15], [16], suggesting that the suppression effect occurs at relatively higher visual processing stages. The earlier ERP components (e.g., P1 and N1) are also suggested to be related to attention but in different ways [12], [17]. Direct comparison of the behavioral and ERP data should provide critical information about the relationship between subjective judgments and each component of neural activity.

Here, we analyzed the ERPs obtained in an experiment reported elsewhere [8], where we focused on the SSVEP data. In the experiment, two sets of flickering stimuli at different frequencies were presented in the left and right visual fields to evoke SSVEPs, and a target was presented at various moments after cueing to a to-be-attended location. Participants had to perform a target detection task in order to keep attention on the instructed location. In the previous report [8], we analyzed the SSVEPs to investigate the time course of attentional modulation after cueing and compared them with that of the target detection performance. We concluded that, with assuming the neural delay as 150 ms, attentional modulation of SSVEP phase coherence rather than SSVEP amplitude corresponded more closely to that of the behavioral performance [8]. The present study further compared different components of ERP elicited by a target with behavioral performance and with SSVEP. We attempted to identify the ERP components in response to the targets presented and then we analyzed the time course of attention from amplitudes of the ERP components as a function of interval between the attention cue and the target onset for each ERP component identified.

The aims of this study were to investigate (1) whether attentional states could be tracked with changes in ERP amplitudes and (2) how the ERPs are related to behavioral performance and concurrently recorded SSVEPs.

## Materials and Methods

The experimental methods are briefly described here, with detailed information given elsewhere [8].

### Participants

Eight participants (1 female; 22–32 years of age) with normal or corrected-to-normal visual acuity took part in the experiment. Data from one participant was excluded due to excessive artifacts in the electro-oculogram (EOG) and the retroactive report of the failure of the experimental task. All participants gave written informed consent. This study was approved by the Ethics Committee of the Research Institute of Electrical Communication, Tohoku University.

### Stimuli

**Figure 1:**
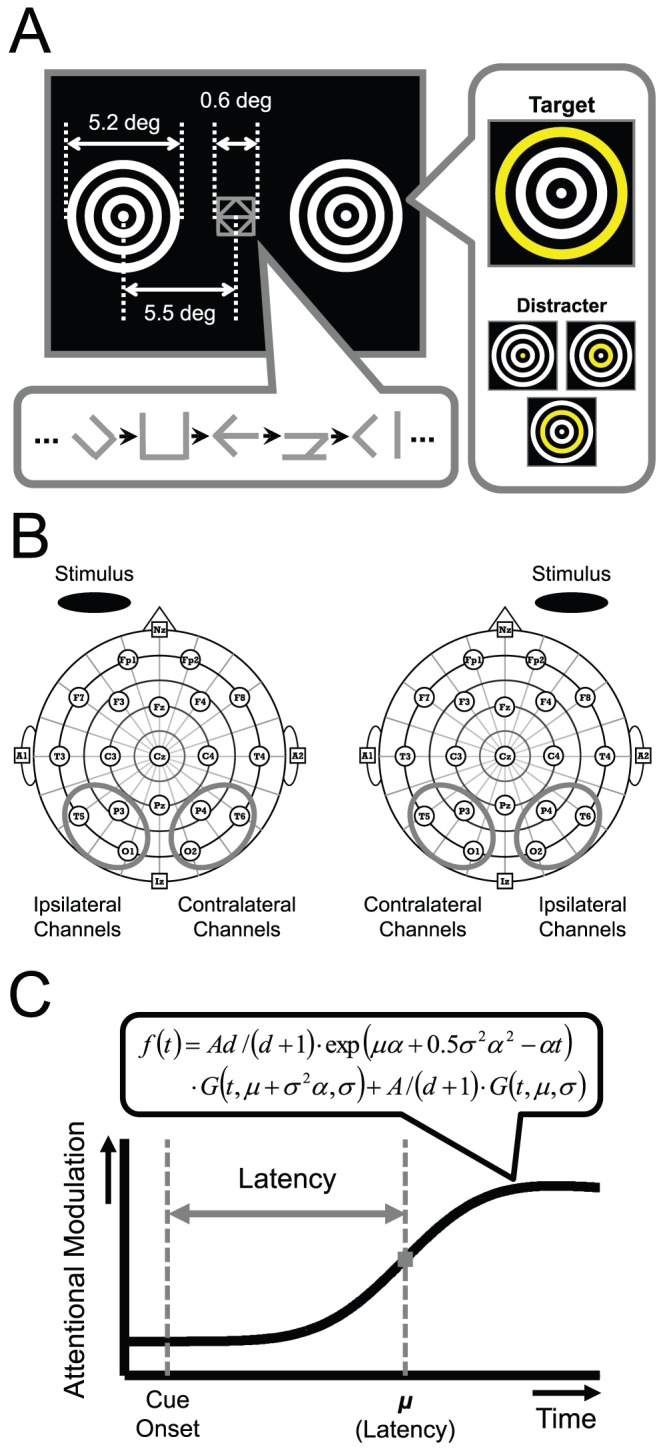
Experimental stimulus and data analysis. (A) Visual stimulus display used in this experiment. Two concentric rings presented on the left and right sides of the center of the display flickered at different temporal frequencies (21.0 and 28.0 Hz). A rapid serial visual presentation stream including an arrow-shaped cue was presented at the center of the display. Participants were instructed to detect the cue, which was presented only once in a trial, and to direct attention to either of the flickering rings indicated by the cue. For a behavioral task, target and distracter patterns were superimposed on the flickering stimulus. Each participant was instructed to respond to color changes that occurred at the outermost ring of the attended side (target) by a button press, and not to do so for the other color changes (distracters) or all color changes on the ignored side. (B) Illustration of the electrode locations. The posterior channels (P3/P4, O1/O2, T5/T6; enclosed by circles) contralateral and ipsilateral to the target stimulus were used for the analysis. (C) The modified cumulative Gaussian function used for time-course analysis. The onset latency of attentional modulation was defined as the parameter “*μ*” of the function.

Figure 1A shows a schematic view of our stimulus. Two circular square-wave gratings (5.2 deg diameter and 1.25 cycles/deg) were presented, centered at 5.5 deg eccentricity on the left and right sides of the central fixation marker. The stimuli were presented against a dark background (<0.1 cd/m^2^). The maximum luminance of the rings was 143 cd/m^2^. To evoke SSVEPs, the luminance of the rings was modulated sinusoidally at 21.0 Hz on one side and at 28.0 Hz on the other. The depth of the luminance modulation was 100% for both stimuli. There were two reasons for using the relatively higher frequencies (21.0 and 28.0 Hz). First, high temporal frequency stimuli realize to measure changes in SSVEP with high temporal resolution. Second, we can differentiate SSVEP signals to high frequency stimuli easily from the effect of the transient signal evoked by the cue presentation. Empirically, the transient EEG contains the relatively lower frequency components (∼10 Hz).

An arrow-shaped stimulus pointing either left or right was presented as a cue at the center of the display to indicate the to-be-attended circular grating.

A randomly chosen ring at each location changed color from white to yellow every 143 ms (7 times/s). The color change at the outmost ring was defined as the target and those at the other rings as distracters. The same target (distracter) pattern was never presented successively. The participant's task was to respond to every target at the attended location. The interval between cue onset and target onset was randomly set with a constraint that the minimum inter-target interval was 429 ms. The total number of attended target presentations after the cue onset (a period of 3 sec) was 4.2 on average in each trial. The range of the number of target presentations per trial was two to six for all participants. The number of targets and distracters in each time-bin was set to 80 and 320, respectively. Note that the actual number of targets and distracters was differed across time-bins and participants because some trials were rejected due to the EOG artifacts or the erroneous report of cue direction.

### Experimental procedure

Each trial was started with a key press by participants. Two sets of stationary circular gratings were presented and they started flickering 500 ms later. Flicker frequencies were randomly assigned to the left and right gratings on each trial (21.0 Hz left and 28.0 Hz right or 28.0 Hz left and 21.0 Hz right). The duration between flicker onset and cue onset was chosen randomly from 1,200, 1,400, 1,600, 1,800, and 2,000 ms on each trial. Participants were instructed to shift their attention toward the grating to which the arrow pointed. The two gratings remained flickering until 3,000 ms after the cue presentation. During this period, participants maintained their attention on the cued grating. Verification of their attention was enforced by a behavioral task. They were instructed to press an assigned key as soon as possible every time they detected a target on the attended side (Figure 1A). Each participant performed some practice trials prior to the EEG recording to be familiarized with the task. The number of the trials was about 20 trials varying across participants.

There were four experimental conditions: two attention locations (left or right) and two flicker-frequency assignments. In each condition, five pre-cue intervals were used and each condition consisted of 100 trials including 20 repetitions of each pre-cue interval.

### EEG recording

We recorded brain electrical activity from 19 scalp electrodes mounted on an elastic cap connected to an EEG recording system (Neurofax EEG-9100, Nihon Koden, Tokyo, Japan). The electrode arrangement was based on the International 10–20 System (Figure 1B). The reference channels were placed on both ear lobes (A1 and A2; see [8] for details). EEG signals were recorded with a band-pass filter of 0.5–120 Hz and digitized at 1,000 Hz. All electrode impedances were confirmed to be below 5 kΩ before each experimental block. Lateral eye movements were also recorded with a bipolar left-to-right outer canthus montage (horizontal EOG) for off-line analysis of the artifacts and were used to exclude trials with EOG deflections of more than ±40 µV from the potential averaged over all data points through the trial, which corresponded approximately to 2.5 deg eye shifts [18]: 5.4% of the trials were judged to be contaminated by eye movement artifacts on average.

**Figure 2:**
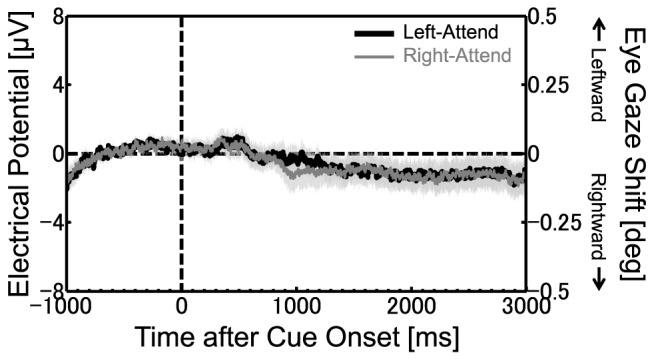
Grand averaged horizontal EOGs time-locked to cue onset. The vertical axes represent the electrical potential and the roughly corresponding eye gaze shift. The positive and negative deflections of EOG indicate the leftward and rightward eye gaze shifts, respectively. The curve shows the time course of EOG when the left stimulus was cued (black line) or when the right stimulus was cued (gray line). Shaded area around each EOG curve shows the standard error across participants (*N* = 7).

To show eye movement control in detail, we averaged the horizontal EOGs across participants (Figure 2). Lines represent the horizontal EOG with the left stimulus cued (black line; left-attend condition) and that with the right stimulus cued (gray line; right-attend condition). There was almost no difference between the left-attend and right-attend conditions, except for the EOG difference corresponding to at most 0.1 deg gaze shift (according to eye-movement measurements after the experiment) around 1,000 ms after the cue onset. This analysis confirmed that the participants reliably maintained their fixation to the central stimulus throughout the trial.

### Analysis

#### Behavioral performance

We evaluated behavioral performance in the target detection task by means of *d′*. Responses in the time window between 199 and 585 ms from each target onset were regarded as hits and the others as false alarms (FAs). This time window covered 99.7% (±3SD) of all responses based on the response-time distribution. The hit (or FA) rate was defined as the ratio of the number of hits (or FAs) to the number of all targets (or distracters) presented in each time bin. To evaluate the attentional modulation on the behavioral performance, we computed *d′* for each time bin with a conventional modification for the hit and FA rates of 0 and 1 [19]: we replaced 0 by *1/(2N)* and 1 by *1-1/(2N)*, where “*N*” represents the target/distracter number.

#### Event-related potentials

We extracted the EEG data between 100 ms before and 565 ms after onset of each target (target-related EEG) or distracter (distracter-related EEG). The target-/distracter-related EEGs were averaged across trials separately for each target/distracter bin. The EEG data were analyzed for targets/distracters presented between 0 to 2,433 ms after cue onset because those presented later did not have time data to compare with the other bins because of stimulus termination. The averaged EEG data were digitally low-pass filtered with a cut-off frequency of 15 Hz. To cancel out any components other than the target-related ERPs, such as the cue-evoked responses, we subtracted the distracter-related ERPs from the target-related ERPs in each condition. We will refer to the ERP data as differences between the target-related and distracter-related ERPs in the following section (except for “Appendix S1 & Figure S1 in File S1”).

**Figure 3:**
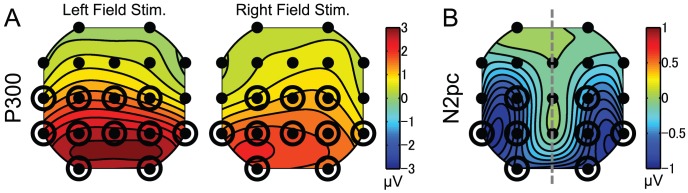
Topographical distribution of attentional modulation on the ERP components. Circles enclosing dots indicate the channels showing significant attentional modulation. (A) Attentional modulation on P300 for left-sided (left) and right-sided (right) stimulus. (B) Attentional modulation on N2pc. The map on left hemisphere represents the N2pc elicited by the target on left visual field, while that on right hemisphere the N2pc by the target on right visual field. The topography was made with setting the N2pc amplitude at midline channels (Fz, Cz and Pz) to zero, because hemisphere differences cannot be defined for the midline channels.

To select channels for the analyses, we analyzed topographical distributions of attentional modulation in the ERP components (Figure 3). The topography data were computed as time-averages of the ERP waveforms to targets presented between 1,000 and 2,433 ms after the cue onset. The time window for the computation of the ERP amplitude for P300 was between 339 and 439 ms after the target onset and that for N2pc was between 190 and 290 ms (See “Results” section for P300 and N2pc identification).

The P300 amplitude was defined as the difference between attended and ignored conditions. Although maximum amplitude of P300 has typically been reported at the mid-line channels of scalp surface (e.g., Pz; see [20] for a review), the P300 topographies show broadly distributed attentional modulations (Figure 3A). We performed three factors ANOVA for P300 data with repeated measures by taking attentional state (attend vs. ignore), stimulus side (left vs. right visual field), and channel (19 sites) as the factors. The significant main effects were found for attentional state [*F*(1,6) = 13.81, *p*<.05] and channel [*F*(18,108) = 13.37, *p*<.05]. The significant interactions were found between attentional state×stimulus side [*F*(1,6) = 6.55, *p*<.05], attentional state×channel [*F*(18,108) = 8.00, *p*<.05], stimulus side×channel [*F*(18,108) = 2.92, *p*<.05], and the three factors [*F*(18,108) = 3.43, *p*<.05]. Analyses of simple main effects revealed that the significant attentional modulation was found at C3, C4, P3, P4, O1, O2, T3, T5, T6, Cz, and Pz for the left-stimulus, and at C3, C4, P3, P4, O1, O2, T5, T6, Cz, and Pz for the right-stimulus (dots enclosed by a circle, all *ps*<.05).

On the other hand, the N2pc topography shows the posterior-lateralized scalp distribution (Figure 3B), as in the previous studies (e.g., [21]). The N2pc amplitude was defined as the difference between the corresponding pair of channels contralateral and ipsilateral to the stimulus (e.g., contralateral O1 and ipsilateral O2). The color map on the left and right hemisphere represents the N2pc amplitude to targets presented in the left and right visual field, respectively. We also performed a repeated-measures ANOVA for the N2pc data with factors of hemisphere (contralateral vs. ipsilateral), stimulus side (left vs. right visual field), and channel set (8 sets: Fp1/Fp2, F3/F4, C3/C4, P3/P4, O1/O2, F7/F8, T3/T4, and T5/T6). The main effects were found to be significant for hemisphere [*F*(1,6) = 9.41, *p*<.05] and stimulus side [*F*(1,6) = 9.03, *p*<.05]. The significant interactions were found between hemisphere×channel set [*F*(7,42) = 7.55, *p*<.05], and between stimulus side×channel set [*F*(7,42) = 9.52, *p*<.05]. Analyses of simple main effects revealed that the significant attentional modulation was found between the channels in the pairs of C3/C4, P3/P4, O1/O2, and T5/T6 (dots enclosed by a circle, all *ps*<.05).

Based on the above-mentioned analyses, we used data from the posterior channels (P3/P4, O1/O2 and T5/T6) for further ERP analyses in the main results. These channels are the same as those we used for SSVEP analyses in the previous study. We collapsed the ERP data across the channels within each hemisphere, contralateral and ipsilateral to the stimulus side (Figure 1B).

**Figure 4:**
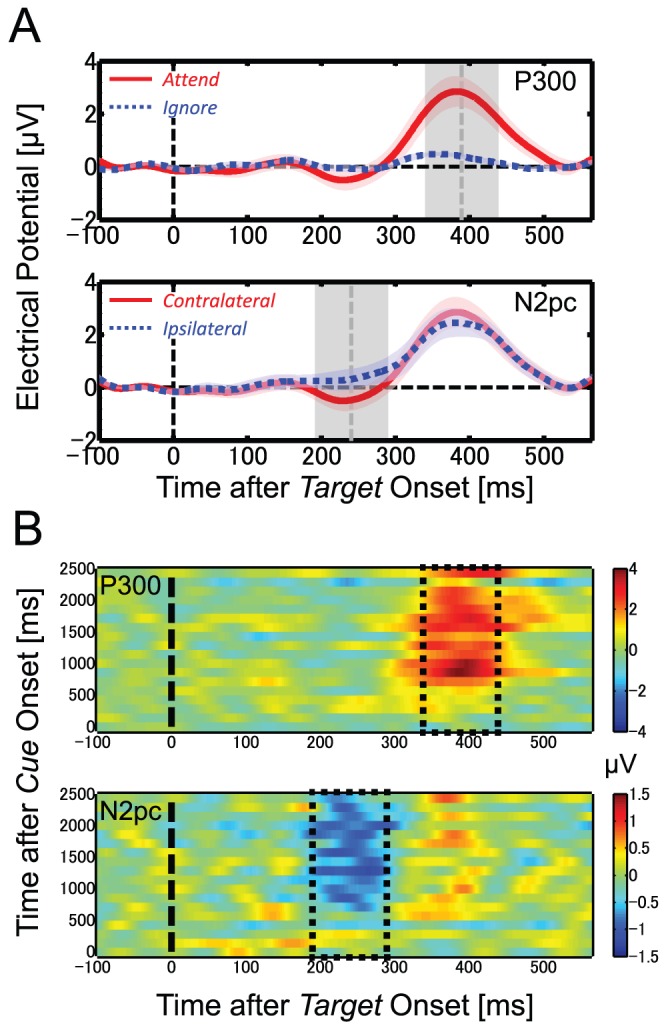
Grand average ERP waveforms time-locked to target onset. The waveform was digitally low-pass filtered with a cut-off of 15 Hz and was baseline-corrected with the mean values between 100 and 1 ms before target onset. Data were obtained from the three pairs of occipito-temporal electrodes (P3/P4, O1/O2, and T5/T6). (A) The P300 component is found when comparing ERPs to the attended and ignored targets in the contralateral channels to the target (upper panel). The N2pc component is found when comparing ERPs from the contralateral and ipsilateral channels to the target at the attended location (lower panel). Shaded area around each ERP curve represents the standard error across participants (*N* = 7). Vertical dashed line (gray) indicates the time with the peak modulation for the target-related ERP component. Shaded area around the time with the peak represents a ±50 ms range. We defined the magnitude of the P300 and N2pc components as an average within the shaded area. (B) Color maps of temporal dynamics of P300 (upper panel) and N2pc (lower panel) components. The P300 component was defined as the difference between ERPs to attended and ignored targets. The N2pc component was defined as the difference between ERPs to attended target from contralateral channels and those from ipsilateral channels. Each waveform was baseline-corrected with the mean values between 100 and 1 ms before target onset. The horizontal axis shows time after target onset and the vertical axis shows time after cue onset. The ERP waveform to the target presented in a moment was plotted in the corresponding row. Rectangular areas framed by dotted lines indicate the time windows for defining the ERP amplitudes.

To estimate the time course of attention shift from the ERP components, we analyzed the data in two steps. First, we defined the attentional modulation in the ERP components. For this purpose, we averaged the ERP data to targets presented between 1,000 and 2,433 ms after cue presentation, during which attentional modulation was expected to be maximum and stable. In the averaged waveform, where the baseline was defined as the mean between 100 and 1 ms before target presentation, we found clear attentional modulation for P300 and N2pc but not for other components (Figure 4; See “Results” section). The P300 component was extracted as the difference between ERPs from the contralateral channels to the attended target and those to the ignored target. The N2pc component was extracted as the difference in the ERP to the attended target between the contralateral and the ipsilateral channels. Second, we defined the magnitude of the P300 and N2pc components as the average of ±50 ms range (shaded area in Figure 4A) centered at the time with the peak value. This index was computed for each target bin, in order to investigate the temporal developments in the amplitude of the P300 and N2pc components after cue onset.

#### Latency estimation

To quantify the latency of attentional modulation, we approximated the time course of the attentional modulation using a modified version of the cumulative Gaussian function ([22]; see Figure 1C). We adopted this function because this can express the gradual decay in attentional modulation after reaching a peak shown in the data. The function is
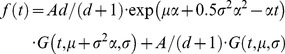
(1)where *t* is the time (ms), *μ* and *σ* are the mean and the standard deviation of the Gaussian function, *α* is the inverse of the time constant of dissipation, *A* is the amplitude of the function, *d* is the ratio of the first term to the second, and *G* is the cumulative Gaussian function. We defined the latency of attentional modulation as the mean of the function (*μ*). The first term determines the temporal decrease in attentional modulation, and the second term the overall shape of the function. The curve fitting was implemented by a least squares method, under the constraint that *A* and *d* be positive except that *A* of the N2pc function be negative. The fitting method was applied to the averaged time-course data over participants. The fitted parameters are summarized in Table 1.

**Table 1:** Parameters of the best-fit function in Equation 1.

	Measure	σ	μ	α	A	d
Behavior	d′	139.3	386.3	2.11×10^−4^	1.85	6.47×10^12^
ERP	P300	202.7	622.1	1.18×10^−3^	3.61	1.42
	N2pc	271.2	762.2	3.81×10^−4^	−1.00	2.23×10^12^
SSVEP	Amplitude	175.1	800.8	1.12×10^−2^	0.313	2.15
	ITPC	123.5	549.3	−1.95×10^−4^	0.087	1.34×10^12^

## Results

### Event-related potentials

#### Identification of ERP components

Figure 4A shows the target-related ERP (actually the difference between the target-related and distracter-related ERPs) waveforms collapsed across targets presented between 1,000 and 2,433 ms after the cue onset. To analyze the P300 component, we compared ERP waveforms to the attended target (red solid line) with that to the ignored target (blue dotted line) recorded from the contralateral posterior channels to the target location (upper panel in Figure 4A). There was a positive deflection in the ERP to the attended target, but no such deflection in the ERP to the ignored target. We defined this positive ERP component as P300 based on the polarity and latency. The peak latency of the P300 was 389 ms after target presentation. To analyze the N2pc component, we compared the ERP waveform to the attended target recorded from the contralateral posterior channels (red solid line) with that from the ipsilateral posterior channels (blue dotted line) (lower panel in Figure 4A). There was a negative deflection in the ERP waveform in the contralateral channels relative to that observed in the ipsilateral channels. We defined this negative ERP component as N2pc. The peak latency of N2pc was 240 ms after target onset. An average over ±50 ms around the peak latency (gray shaded areas in Figure 4A) was calculated as an index of the attentional modulation for each of the P300 and N2pc components.

To show how well the temporal windows capture the ERP components, we plotted time courses of EEG amplitude as functions of time after target onset and time after cue onset (Figure 4B). The figure shows little ERP components outside the window independently of time after target onset and of time after cue onset. This justifies our choice of the temporal windows for the components in the present experiment although there may be a particular ERP component at different time in some cases due to attentional influence on its latency (e.g., [20], [23]).

#### Time-course analysis

**Figure 5:**
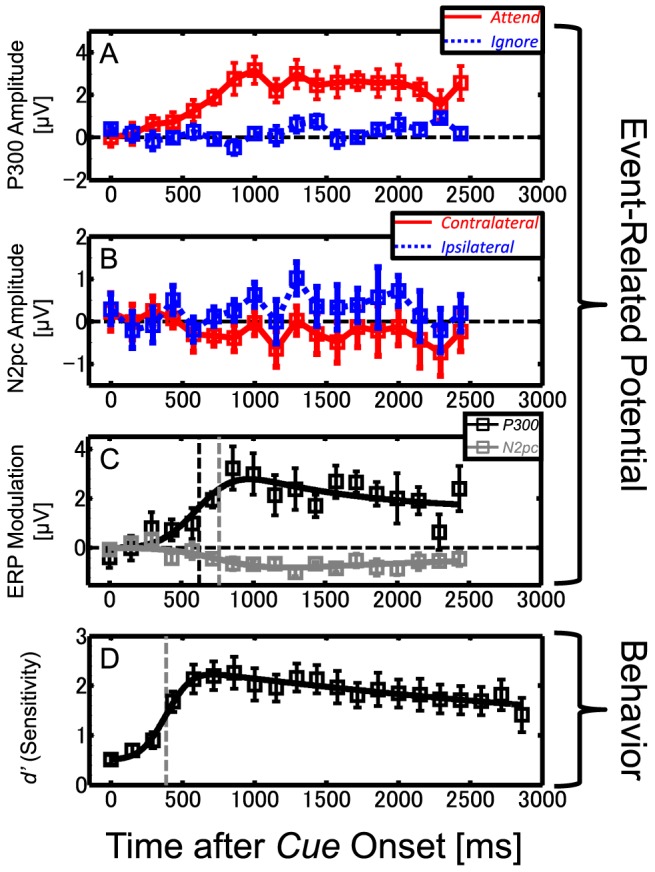
Time-course analysis of ERP and behavioral data. (A, B) Time course of amplitude for P300 (A) and N2pc (B) as a function of time after cue onset, averaged across all participants. Each data point was the temporal average across a ±50 ms range around the time with the peak (vertical shaded areas in Figure 4A). (C) Time course of ERP modulation after cue onset. Each data point was computed as the difference between attended and ignored conditions for P300 (black square) or between contralateral and ipsilateral conditions for N2pc (gray square). Error bar represents the standard error across participants (*N* = 7). Solid lines represent the modified cumulative Gaussian function fitted to the actual data. Vertical dashed lines mark the latency (*μ*) of the fitted function. (D) Time course of the behavioral performance (*d′*) averaged across all participants. Symbols with error bars plotted in each time bin represent averaged data and standard errors across participants (*N* = 7). Solid line shows the function fitted to the actual data. A vertical dashed line marks the latency (*μ*). The behavioral data are same as those in our previous study [8].

Figure 5A shows the time course of the P300 amplitudes averaged across participants for the attended (red solid line) and ignored (blue dotted line) conditions. The P300 amplitudes were subjected to a repeated-measures ANOVA with the factors of attentional state (attend vs. ignore) and time (18 time bins). The results revealed significant effects of attentional state [*F*(1,6) = 20.16, *p*<.05], time [*F*(17,102) = 3.69, *p*<.05], and their interaction [*F*(17,102) = 3.31, *p*<.05]. Analysis of the simple main effects revealed that attentional modulation of the P300 amplitude was significant for targets presented at 717 ms (6th bin) or later except at 2,292 ms (17th bin) after the cue onset (all *ps*<.05). Likewise for the N2pc amplitudes, Figure 5B shows the averaged time course of N2pc amplitudes for the contralateral (red solid line) and ipsilateral (blue dotted line) conditions. The N2pc amplitudes were subjected to a two-factor ANOVA with the factors of hemisphere (contralateral vs. ipsilateral) and time (18 time bins). The analysis revealed a main effect of hemisphere [*F*(1,6) = 12.83, *p*<.05] and an interaction of the two factors [*F*(17,102) = 3.04, *p*<.05], but not a main effect of time [*F*(17,102) = 0.53, *p* = .93, *n.s*]. Analysis of the simple main effects revealed that attentional modulation of the N2pc amplitude was significant for targets presented at 858 ms (7th bin) or later except at 2,433 ms (18th bin) after the cue onset (all *ps*<.05).

Figure 5C shows the difference in P300 between the attended and ignored conditions (black symbols) and the difference in N2pc between the contralateral and ipsilateral conditions (gray symbols). Attentional modulation was larger and appeared to start earlier in the case of the P300 component. To quantify the time courses of attentional modulation, we fitted the modified cumulative Gaussian function (Eq. 1) to each averaged time-course data (solid curves in Figure 5C). For the P300 amplitude, the parameters *μ* and *σ* were estimated as 622 ms and 203 ms, respectively; for the N2pc amplitude, they were estimated as 762 ms and 271 ms, respectively. See Table 1 for the other parameters.

### Behavioral performance

Figure 5D shows the time course of *d′* for target detection after onset of the attention cue. To quantify the latency of the attention shift, we fitted the modified cumulative Gaussian function (Eq. 1) to the baseline-corrected *d′* data (the *d′* value at *t* = 0 msec as the baseline of the function). The solid line depicts the fitted curve. The parameters *μ* and *σ* were estimated as 386 ms and 139 ms, respectively. See Table 1 for the other parameters.

In order to examine the effect of target presentation order, we computed the hit rate for the targets presented at the first time after cuing and that for the second targets in the same time period. We analyzed the data from the 5th time bin (575 ms after cue onset), where the number of the first targets and of the second targets was similar (38.7 and 41.3 on average for the first and second targets, respectively). The results showed no significant difference between the detection performances to the first (54.6%) and second (62.2%) targets [*t*(6) = −1.65, *p* = .15, *n.s*]. We, therefore, did not analyze further the effect of target presentation order in this report.

## Discussion

The aim of this study was to investigate the time course of visual attention using ERP measurements. We found that two ERP components, P300 and N2pc, were modulated by attention. The amplitude of these two ERP components increased with time up to about 1,000 ms and remained approximately constant in the later phase of the trial. These sustained temporal characteristics suggest that amplitude changes follow the temporal dynamics of endogenous attention, and indicate that the amplitude of both the P300 and N2pc components are sensitive measures for attention.

To compare the time course of P300 and N2pc with that of the behavioral performance, we compared their latencies (*μ*) obtained from Eq. 1. The estimated latency was about 620 ms for P300 and 760 ms for N2pc, whereas that of behavioral performance was about 390 ms. If we consider a target presentation of about 140 ms, the estimated latency should have an interval of 140 ms: 390–530 ms for behavioral performance, 620–760 ms for P300, and 760–900 ms for N2pc. The comparison revealed that the latency of both P300 and N2pc was much slower than that of behavioral performance, indicating that behavioral performance was determined by neural activities not reflected by either P300 or N2pc. These results appear to contradict the known effects of attention on P300 and N2pc. Although the P300 component has been suggested to be modulated by several cognitive factors such as working memory, expectation, and awareness, attentional allocation strongly influences P300 amplitude (for a review see [20]). The N2pc component, on the other hand, has been suggested to reflect the orienting of attention to the stimulus location [21], [24]. The apparent contradiction could be solved if we assume that the three measures reflect different aspects of attentional modulation. The late onset of attentional modulation of the P300 component (Figure 5A and C) may reflect brain activities related to visual awareness after target detection, and the further later onset of the N2pc modulation (Figure 5B and C) may reflect attentional orienting to the target location accompanied by target detection. In contrast, the behavioral performance likely corresponds to attentional modulation of the target detection mechanisms. Our time-course measurements thus far have revealed that attentional modulation of behavioral performance differs from that indexed by either P300 or N2pc.

**Figure 6:**
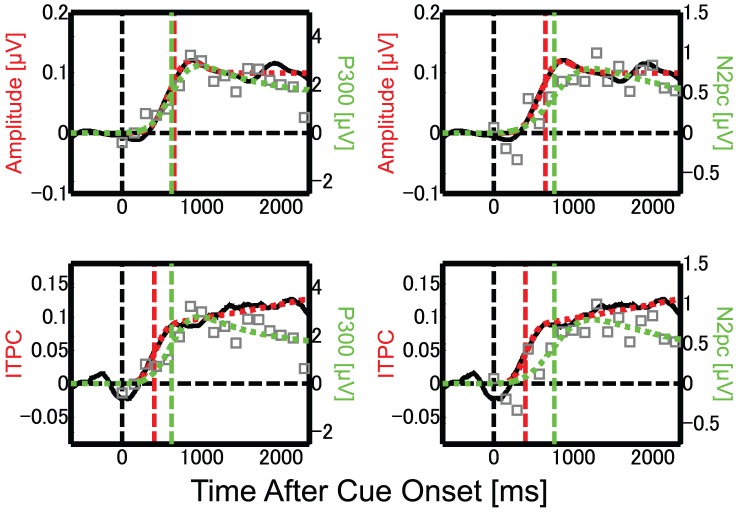
Comparisons of the time-course data. Comparisons of the time courses of attentional modulation in the ERP data and SSVEP data (re-plotted from [8]). The SSVEP amplitude and ITPC are plotted in the upper and lower rows, respectively, indicated by black lines (actual data) and red lines (fitted data). The P300 and N2pc components are plotted in the left and right columns, respectively, by gray squares (actual data) and green lines (fitted data). Vertical line on each function indicates the latency of attentional modulation for each EEG measure. Note that the time course and latency of SSVEP measures were corrected by shifting the function by 150 ms. See the main text for details.

To investigate the relationship between different EEG measurements for attention shifts, we compared the ERP data with the SSVEP results analyzed in our previous study [8]. We compared two SSVEP measures, amplitude and inter-trial phase coherence (ITPC), with the ERP and behavioral data. The SSVEP amplitude is the amplitude in the frequency component of EEG corresponding to the flickering stimulus. The ITPC is an index of phase coherence in neural activity that is calculated from phase variation of the flicker frequency component across trials. The ITPC ranges between 0 and 1, and a higher ITPC indicates a greater degree of phase coherence [8], [25], [26], [27]. The latencies of attention shift estimated from the SSVEPs are summarized in Table 1. As mentioned in the Introduction, we must take into account the time lags between stimulus onset and brain activities evoked by the stimulus when comparing the SSVEP data with the ERP or behavioral data, which are considered to be locked to the latency of target presentation with respect to the cue onset. Here, we assumed the lag for SSVEP as 150 ms following previous studies [6], [12]. The corrected latency is 650 ms for the SSVEP amplitude and 400 ms for ITPC after the subtraction of 150 ms from each original latency value. The time courses of attentional modulation for each ERP component and for the corrected SSVEP are shown in Figure 6. The corrected latency of the SSVEP amplitude was similar to the latency of P300 (620–760 ms), while the corrected latency of ITPC was similar to the latency of behavioral performance (390–530 ms). Neither was close to the latency of N2pc (760–900 ms). These findings indicate that behavioral performance in the target detection task is determined by the earlier stages of visual processing among the multiple stages of attentional modulation, and that the stage could be reflected in ITPC but not in either the P300 or N2pc components.

Since the P300 and N2pc components were elicited by the targets requiring the behavioral responses, one might expect that they are related to behavioral performance more closely than SSVEP, which was irrelevant to the task. However, attentional modulation has been found at various stages of visual system, including very early stages such as V1 [28], [29], and the task-irrelevant SSVEPs could reflect the neural activities determining the task performance. Although other ERP components such as P1 and N1 may be more closely related to behavioral performance (e.g., [30]), the components were not found in the present experiment (Figure 4).

In this study, the ERP analyses showed clear P300 and N2pc components, but not the other ERP components which are thought to be related to attention such as P1 and N1. A previous study using a similar paradigm reported the attentional modulation of the early ERP components of P1, N1, and N2, as well as P300 (called P3 in their study) [31]. They also found significant correlations in the data of individual participants between attentional modulations of the SSVEP amplitudes and of the N1 and N2 components, but not of the P1 and P3 components. Consistent with their results, our comparisons of the time-course data provide clear evidence for different attentional mechanisms contributing to the different EEG measures. Taken together with the findings of previous reports, we conclude that SSVEPs are closely related to behavioral performance [6], [8], [9], [32], [33] and the ERP components of N1 and N2 [31].

Several studies have shown the (at least partially) dissociated neural generators for P300, N2pc and SSVEP. The P3b component, which is likely the same as P300 in the present study, is localized in temporal-parietal areas [20], while the N2pc component is thought to be generated in parietal and occipito-temporal cortices [34]. The neural loci of SSVEP, on the other hand, have been identified at early visual areas including V1, V2, and V3 [35], [36], [37], [38]. In sum, the present results with the above-mentioned studies support our conclusion in the previous study that the behavioral performance is determined by attentional modulation of neural response coherence at early visual cortical areas ([8]; see also [26]). It must be noted that tasks and conditions could change the neural stage of attentional modulation that determines the behavioral performance (e.g., [35], [39]).

Note that we could not analyze the P1, N1 and N2 components simply because they were not elicited under our experimental conditions. A previous study using similar stimuli showed these ERP components with clear attentional modulation [31]. The reason for this lack of elicitation is not clear, but there is one possible reason: the target stimulus (color change on the outmost ring) was too weak relative to the flicker stimulations, and the SSVEPs might have masked the ERP components. If so, our results may indicate that the SSVEPs did not mask the later ERP components of P300 and N2pc. This is consistent with the notion that P300 and N2pc are different from SSVEP in their neural sources.

It should be noted that the behavioral performance showed a systematic bias, as the *d′* data was clearly above chance (*d′* = 0) at the time of cue onset. A recent study has pointed out a problem in defining the denominator of FA rate, in the experimental paradigms with high target (distracter) presentation rates [40]. We defined the number of distracters as the denominator, which could underestimate the FA rate. This could in turn overestimate the whole *d′* values. Nonetheless, we decided not to adopt the correction procedure by [40] for the “overestimated” *d′* for the following reasons. First, the procedure was not simply applicable to our time-course analysis, because the corrected denominator was computed for the total number of FAs for the numerator, so that the correction of the FA rate for particular time period cannot be made. Second, the primary purpose of this paper is to compare the previously published time-course data (behavioral performance and SSVEP) with the ERP data. Therefore the behavioral data should be identical to our previous data. Third, the systematic bias in behavioral performance likely raises the whole level of the *d′* time-course data. We corrected for the baseline in the *d′* data to estimate the time course of behavioral performance, which should cancel out the influence of the overestimation.

We discussed the relationship between behavioral performance and neural activity based on the data during attention shift (see also [6], [7]). However evidence for (or against) the link has also come from the data during sustained attention (e.g. [35], [39], [41]). These studies have explored the correlation (or dissociation) between behavioral performance and neural activity across various stimulus/task conditions when attention is fully engaged in the stimulus/task. Combining the two methodologies will, perhaps, provide clearer pictures about the relationship between behavior and physiology in future studies.

In summary, we showed that amplitude of the two ERP components P300 and N2pc was sensitive to the attentional state and was useful for the time-course analysis of attention. The latency of attention shift estimated from these ERP components was longer than that estimated from the behavioral performance, indicating that attentional modulation of target detection was determined at earlier processing stages than the stages in which these ERP components were related. Future research should investigate exactly how the ERP components are related to behavioral performance. The technique for measuring the temporal dynamics of ERPs developed in this study could be an important tool for investigating the different stages of attention-related brain activities.

## Supporting Information

File S1 Appendix S1 & Figure S1. The time courses of target-related and distracter-related ERP amplitudes as a function of time after cue onset.
(PDF)

## References

[1] Posner MI (1980) Orienting of attention. Q J Exp Psychol 32: 3–25. 7367577 10.1080/00335558008248231

[2] Posner MI, Snyder CR, Davidson BJ (1980) Attention and the detection of signals. J Exp Psychol 109: 160–174. 7381367

[3] Egeth HE, Yantis S (1997) Visual attention: control, representation, and time course. Annu Rev Psychol 48: 269–297. 9046562 10.1146/annurev.psych.48.1.269

[4] Morgan ST, Hansen JC, Hillyard SA (1996) Selective attention to stimulus location modulates the steady-state visual evoked potential. Proc Natl Acad Sci U S A 93: 4770–4774. 8643478 10.1073/pnas.93.10.4770 PMC39354

[5] Müller MM, Picton TW, Valdes-Sosa P, Riera J, Teder-Sälejärvi WA, et al (1998) Effects of spatial selective attention on the steady-state visual evoked potential in the 20–28 Hz range. Brain Res Cogn Brain Res 6: 249–261. 9593922 10.1016/s0926-6410(97)00036-0

[6] Müller MM, Teder-Sälejärvi W, Hillyard SA (1998) The time course of cortical facilitation during cued shifts of spatial attention. Nat Neurosci 1: 631–634. 10196572 10.1038/2865

[7] Andersen SK, Müller MM (2010) Behavioral performance follows the time course of neural facilitation and suppression during cued shifts of feature-selective attention. Proc Natl Acad Sci U S A 107: 13878–13882. 20643918 10.1073/pnas.1002436107 PMC2922290

[8] Kashiwase Y, Matsumiya K, Kuriki I, Shioiri S (2012) Time Courses of Attentional Modulation in Neural Amplification and Synchronization Measured with Steady-state Visual-evoked Potentials. J Cogn Neurosci 24: 1779–1793. 22360591 10.1162/jocn_a_00212

[9] Müller MM (2008) Location and features of instructive spatial cues do not influence the time course of covert shifts of visual spatial attention. Biol Psychol 77: 292–303. 18083290 10.1016/j.biopsycho.2007.11.003

[10] Hayden BY, Gallant JL (2005) Time course of attention reveals different mechanisms for spatial and feature-based attention in area V4. Neuron 47: 637–643. 16129394 10.1016/j.neuron.2005.07.020

[11] Herrington TM, Assad JA (2010) Temporal sequence of attentional modulation in the lateral intraparietal area and middle temporal area during rapid covert shifts of attention. J Neurosci 30: 3287–3296. 20203188 10.1523/JNEUROSCI.6025-09.2010 PMC2854171

[12] Hillyard SA, Anllo-Vento L (1998) Event-related brain potentials in the study of visual selective attention. Proc Natl Acad Sci U S A 95: 781–787. 9448241 10.1073/pnas.95.3.781 PMC33798

[13] Sergent C, Baillet S, Dehaene S (2005) Timing of the brain events underlying access to consciousness during the attentional blink. Nat Neurosci 8: 1391–1400. 16158062 10.1038/nn1549

[14] Woodman GF, Luck SJ (1999) Electrophysiological measurement of rapid shifts of attention during visual search. Nature 400: 867–869. 10476964 10.1038/23698

[15] Luck SJ, Vogel EK, Shapiro KL (1996) Word meanings can be accessed but not reported during the attentional blink. Nature 383: 616–618. 8857535 10.1038/383616a0

[16] Vogel EK, Luck SJ, Shapiro KL (1998) Electrophysiological evidence for a postperceptual locus of suppression during the attentional blink. J Exp Psychol Hum Percept Perform 24: 1656–1674. 9861716 10.1037//0096-1523.24.6.1656

[17] Hillyard SA, Vogel EK, Luck SJ (1998) Sensory gain control (amplification) as a mechanism of selective attention: electrophysiological and neuroimaging evidence. Philos Trans R Soc Lond B Biol Sci 353: 1257–1270. 9770220 10.1098/rstb.1998.0281 PMC1692341

[18] Luck SJ (2005) An introduction to the event-related potential technique. Cambridge: MIT Press.

[19] Macmillan NA, Kaplan HL (1985) Detection theory analysis of group data: estimating sensitivity from average hit and false-alarm rates. Psychol Bull 98: 185–199. 4034817

[20] Polich J (2007) Updating P300: an integrative theory of P3a and P3b. Clin Neurophysiol 118: 2128–2148. 17573239 10.1016/j.clinph.2007.04.019 PMC2715154

[21] Luck SJ, Hillyard SA (1994) Spatial filtering during visual search: evidence from human electrophysiology. J Exp Psychol Hum Percept Perform 20: 1000–1014. 7964526 10.1037//0096-1523.20.5.1000

[22] Khayat PS, Spekreijse H, Roelfsema PR (2006) Attention lights up new object representations before the old ones fade away. J Neurosci 26: 138–142. 16399680 10.1523/JNEUROSCI.2784-05.2006 PMC6674304

[23] Brisson B, Robitaille N, Jolicoeur P (2007) Stimulus intensity affects the latency but not the amplitude of the N2pc. Neuroreport 18: 1627–1630. 17885614 10.1097/WNR.0b013e3282f0b559

[24] Luck SJ, Hillyard SA (1994) Electrophysiological correlates of feature analysis during visual search. Psychophysiology 31: 291–308. 8008793 10.1111/j.1469-8986.1994.tb02218.x

[25] Ding J, Sperling G, Srinivasan R (2006) Attentional modulation of SSVEP power depends on the network tagged by the flicker frequency. Cereb Cortex 16: 1016–1029. 16221931 10.1093/cercor/bhj044 PMC1880883

[26] Kim YJ, Grabowecky M, Paller KA, Muthu K, Suzuki S (2007) Attention induces synchronization-based response gain in steady-state visual evoked potentials. Nat Neurosci 10: 117–125. 17173045 10.1038/nn1821

[27] Schack B, Klimesch W (2002) Frequency characteristics of evoked and oscillatory electroencephalic activity in a human memory scanning task. Neurosci Lett 331: 107–110. 12361852 10.1016/s0304-3940(02)00846-7

[28] Kanwisher N, Wojciulik E (2000) Visual attention: insights from brain imaging. Nat Rev Neurosci 1: 91–100. 11252779 10.1038/35039043

[29] Ress D, Backus BT, Heeger DJ (2000) Activity in primary visual cortex predicts performance in a visual detection task. Nature Neuroscience 3: 940–945. 10966626 10.1038/78856

[30] Hopfinger JB, Mangun GR (1998) Reflexive attention modulates processing of visual stimuli in human extrastriate cortex. Psychological Science 9: 441–446. 26321798 10.1111/1467-9280.00083 PMC4552358

[31] Müller MM, Hillyard S (2000) Concurrent recording of steady-state and transient event-related potentials as indices of visual-spatial selective attention. Clin Neurophysiol 111: 1544–1552. 10964063 10.1016/s1388-2457(00)00371-0

[32] Attar CH, Andersen SK, Müller MM (2010) Time course of affective bias in visual attention: Convergent evidence from steady-state visual evoked potentials and behavioral data. Neuroimage 53: 1326–1333. 20615472 10.1016/j.neuroimage.2010.06.074

[33] Müller MM, Andersen SK, Keil A (2008) Time course of competition for visual processing resources between emotional pictures and foreground task. Cereb Cortex 18: 1892–1899. 18063562 10.1093/cercor/bhm215

[34] Hopf JM, Luck SJ, Girelli M, Hagner T, Mangun GR, et al (2000) Neural sources of focused attention in visual search. Cerebral Cortex 10: 1233–1241. 11073872 10.1093/cercor/10.12.1233

[35] Andersen SK, Hillyard SA, Müller MM (2008) Attention facilitates multiple stimulus features in parallel in human visual cortex. Curr Biol 18: 1006–1009. 18595707 10.1016/j.cub.2008.06.030

[36] Di Russo F, Pitzalis S, Aprile T, Spitoni G, Patria F, et al (2007) Spatiotemporal analysis of the cortical sources of the steady-state visual evoked potential. Hum Brain Mapp 28: 323–334. 16779799 10.1002/hbm.20276 PMC6871301

[37] Hillyard SA, Hinrichs H, Tempelmann C, Morgan ST, Hansen JC, et al (1997) Combining steady-state visual evoked potentials and fMRI to localize brain activity during selective attention. Hum Brain Mapp 5: 287–292. 20408230 10.1002/(SICI)1097-0193(1997)5:4<287::AID-HBM14>3.0.CO;2-B

[38] Müller MM, Andersen S, Trujillo NJ, Valdes-Sosa P, Malinowski P, et al (2006) Feature-selective attention enhances color signals in early visual areas of the human brain. Proc Natl Acad Sci U S A 103: 14250–14254. 16956975 10.1073/pnas.0606668103 PMC1599943

[39] Andersen SK, Fuchs S, Müller MM (2011) Effects of feature-selective and spatial attention at different stages of visual processing. J Cogn Neurosci 23: 238–246. 19702461 10.1162/jocn.2009.21328

[40] Bendixen A, Andersen SK (2013) Measuring target detection performance in paradigms with high event rates. Clin Neurophysiol 124: 928–940. 23266090 10.1016/j.clinph.2012.11.012

[41] Andersen SK, Müller MM, Hillyard SA (2009) Color-selective attention need not be mediated by spatial attention. J Vis 9: 2 1–7. 10.1167/9.6.2 19761293

