# Supplementary material for: Temporal Dynamics of Visual Attention Measured with Event-Related Potentials
Source: PLoS One. 2013 Aug 19;8(8):e70922. doi: 10.1371/journal.pone.0070922 (PMC3747140; doi:10.1371/journal.pone.0070922)
Supplement: File S1 — Appendix S1 & Figure S1. The time courses of target-related and distracter-related ERP amplitudes as a function of time after cue onset. (PDF) [file pone.0070922.s001.pdf]

Temporal Dynamics of Visual Attention Measured with Event-Related Potentials

Yoshiyuki Kashiwase, Kazumichi Matsumiya, Ichiro Kuriki, and Satoshi Shioiri

Appendix S1

The ERP amplitudes were computed as the difference between the target-related and the distracter-related ERP amplitudes. One might argue that the subtraction procedure masked or introduce differences of the ERP components. Figure S1 shows the time course of P300 (upper) and N2pc (lower) amplitude to targets (black solid line) and distracters (gray dotted line) separately. The time course of P300 was the difference between attended and ignored conditions, while that of N2pc was the difference between contralateral and ipsilateral conditions. Figure S1 shows that there were some amplitude changes in the time courses of the distracter-related ERP amplitudes. However, they are likely the transient deflections of ERP amplitude immediately after cue onset because the same trend is seen in the target-related ERPs and these deflections are irrelevant to the target processing. We adopted the subtraction procedure because it canceled out the influence of transient responses evoked.

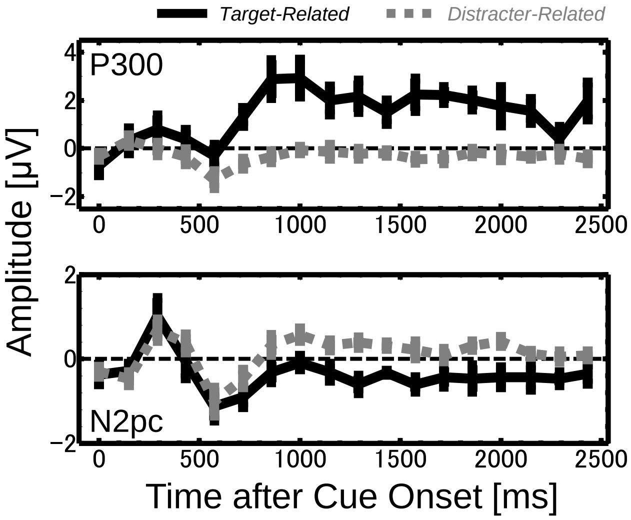

Figure S1. The time courses of target-related and distracter-related ERP amplitudes as a function of time after cue onset. The upper and lower panels show the time courses of P300 and N2pc

components, respectively. The target-related and distracter-related ERP amplitudes are shown by black solid line and gray dotted line, respectively. Each error bar represents the standard error across participants (*N*=7).
